# Strategy and Efficacy of Surgery for Congenital Cervicothoracic Scoliosis with or without Hemivertebra Osteotomy

**DOI:** 10.1111/os.13480

**Published:** 2022-08-30

**Authors:** Hong‐qi Zhang, Yu‐xuan Du, Jin‐yang Liu, Ang Deng, Jian‐huang Wu, Yu‐xiang Wang, Chao‐feng Guo

**Affiliations:** ^1^ Department of Spine Surgery and Orthopaedics Xiangya Hospital of Central‐South University Changsha China

**Keywords:** Cervicothoracic scoliosis, Congenital scoliosis, Correction surgery, Fusion surgery, Osteotomy

## Abstract

**Objective:**

Cervicothoracic scoliosis will cause severe deformities in the early stage, and its structure is complex and the surgical methods are varied. The purpose of this research is to explore the indication and analyze the corrective effect of the two different posterior approach surgical strategies, including correction with fusion and hemivertebra osteotomy, for congenital cervicothoracic scoliosis deformities in children and adolescents.

**Methods:**

This was a retrospective study of 21 patients with cervicothoracic scoliosis who received surgical treatment from January 2010 to June 2020, including nine cases of posterior hemivertebra osteotomy and fusion surgery and 12 cases of posterior correction and fusion alone. The Cobb angle, T1 tilt angle, clavicular angle, neck tilt angle, radiographic shoulder height, sagittal vertical axis, coronal balance distance, and local kyphosis angle were measured preoperatively, postoperatively, and at the last follow‐up. Posterior approach hemivertebra resection or correction with fusion surgery was adopted based on the different individual characteristics of deformity such as main curve Cobb angle, growth potential, and flexibility. Patients were divided into two groups (osteotomy group and nonosteotomy group) according to whether a hemivertebra osteotomy was performed, and the corrective results in the two groups were compared. Paired‐sample *t* tests or independent‐sample *t* tests were used.

**Results:**

The median follow‐up after surgery of the 21 patients was 36 months (range, 18–72 months). The Cobb angle was corrected from 45.81° ± 14.23° preoperatively to 10.48° ± 5.56° postoperatively (correction rate, 77.78% ± 8.93%). The T1 tilt angle decreased from 15.26° ± 7.08° preoperatively to 3.33° ± 2.14° postoperatively (correction rate,73.42% ± 21.86%). The radiographic shoulder height was corrected from 1.13 ± 0.74 cm preoperatively to 0.52 ± 0.42 cm postoperatively (correction rate, 39.51% ± 35.65%). The clavicular angle improved from 2.52° ± 1.55° preoperatively to 1.16° ± 0.96° postoperatively (correction rate, 47.18% ± 35.84%). No significant differences were found at the last follow‐up (*p* > 0.05). The Cobb angle of the main curve, T1 tilt angle, clavicular angle, cervical tilt angle, and shoulder height difference were similar in the two groups (*p* > 0.05).

**Conclusions:**

Posterior approach hemivertebra resection or correction with fusion surgery can be used in the treatment of congenital cervicothoracic scoliosis with satisfactory results, and the surgeon can make an individualized surgical plan according to individual characteristics of deformity.

## Graphical abstract

A 12‐year‐old boy with C7 hemivertebra and multiple malformations in the lower spine underwent a C7 hemivertebra osteotomy and internal fixation from C5‐T3 *via* one‐stage posterior surgery. (A) preoperative X‐ray; (B) preoperative CT; (C, D) The C7 hemivertebra was resected by posterior approach surgery; (E, F) X‐ray at the 2‐year follow‐up.

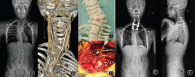

## Introduction

Congenital cervicothoracic scoliosis (CTS) refers to spinal deformities occurring from T4 to C4.^1^ Located at the junction of the relatively fixed thoracic spine and flexible cervical spine, close to the shoulders, deformities in this segment cause an obvious imbalance in the shoulders and neck that can develop rapidly and even cause facial asymmetry. However, conservative treatment, such as plaster fixation or orthopaedic brace fixation, has little effect. Therefore, if cervical and thoracic spinal malformations are clearly diagnosed and there is an obvious abnormal appearance, surgery should be performed early to avoid further aggravation and difficulty in subsequent treatment.^2^ Meanwhile, deformities in the transitional area of the flexible cervical spine and fixed thoracic spine often lead to difficulties with and even the failure of internal fixation.

The cervicothoracic spine has a complex anatomical structure^3^, ^4^ and is located near important neurovascular structures. The aortic arch and superior vena cava are in front of the upper thoracic segment, while the lower cervical vertebrae are adjacent to the brachiocephalic trunk and carotid arteries. There are also important blood vessels, such as the vertebral arteries, in the transverse foramen. Damage to important blood vessels can lead to intraoperative hemorrhage, postoperative hematoma and other complications. Sympathetic nerve injury, which leads to Horner syndrome, has also been reported in cervicothoracic surgery, and most of these injuries are caused by the osteotomy process. The C7‐T1 nerve roots participate in the formation of the brachial plexus, and if they are damaged, sensorimotor disorders of the upper limbs can occur, which greatly increase the difficulty and risk of hemivertebra resection above T1 and C7. To reduce the surgical risk while correcting the deformity, a detailed surgical plan should be developed preoperatively. The surgical strategy for CTS varies widely^5^, ^6^, ^7^, ^8^, ^9^, with various corrective effects and rare reports. Some scholars believe that posterior approach or combined anterior and posterior approach three column osteotomy surgery is the fundamental method to treat CTS, while others believe that the osteotomy surgery of the anterior column of the spine might be avoided through preoperative traction.

This study aimed (i) to study the methods and efficacy of posterior approach correction surgery for CTS, (ii) to compare the corrective effects and follow‐up results between one‐stage posterior approach correction surgery with and without hemivertebraosteotomy, and (iii) to explore an individualized surgical plan according to individual characteristics of deformity. According to individual differences in deformity, different correction methods were adopted to correct the malformations with one‐stage posterior approach surgery, and satisfactory therapeutic effects were achieved, as reported below.

## Materials and Methods

### Inclusion and Exclusion Criteria for Patients

The inclusion criteria were as follows: (i) hemivertebra or scoliosis apex in the cervicothoracic spine (C6‐T4); (ii) treatment with one‐stage posterior approach surgery; and (iii) follow‐up for more than 1 year. The exclusion criteria were as follows: history of spinal surgery; incomplete imaging data or unqualified appearance; and previous anterior correction surgery with internal fixation or multiple correction operations, such as combined anterior and posterior approach surgery (Table S1).

### General Information

A total of 21 patients with congenital CTS admitted to our hospital from January 2010 to June 2020 were included, including seven male patients and 14 female patients, aging from 7 to 19 years.

The apex of scoliosis was located at C7 in two cases, T1 in one case, T2 in three cases, T3 in six cases, and T4 in nine cases. The Cobb Angle of all patients were ranging from 22.40° to 93.20°, with an average of 45.81° ± 14.23°. The Risser sign ranged from 0 to 5, with an average of 3.38 ± 1.49.

This study has been approved by the Ethics Committee of Xiangya Hospital, Central South University (ethics approval number: 201703359).

### Preoperative Examination

X‐ray, computed tomography (CT), and magnetic resonance imaging (MRI) of the full length of the spine were performed to determine the type and location of the malformation, and bilateral bending of the spine and lateral cervical flexion were examined radiographically to clarify the spinal flexibility. CT angiography (CTA) was performed to determine the path of the carotid arteries, aortic arch, vertebral arteries, and other important blood vessels and to determine if there were any vascular malformations. Electroneuromyography was performed to rule out neurological abnormalities and as training for intraoperative arousal experiments.

### Surgical Methods

All patients underwent surgery after giving informed consent. Intraoperative neurophysiological monitoring was performed to indicate intraoperative changes in nerve function, and an arousal test was used to assess spinal nerve function after correction.

For patients with good flexibility (a decrease in the main curve Cobb angle of more than 25% in the lateral bending position), relatively scattered and long scoliosis segments, or with satisfactory traction effect and posterior correction with fusion were selected. For patients with poor flexibility (decrease in main curve Cobb angle of less than 25% in the lateral bending position), large Cobb Angle, short and sharp scoliosis segments, and significant growth potential, posterior approach hemivertebra osteotomy should be selected.

In this study, nine patients underwent hemivertebra osteotomy and the remaining 12 patients underwent posterior correction fusion. Among them, the Cobb Angle of nine hemivertebra osteotomy patients was 48.73° ± 9.15°, flexibility ratios of scoliosis segment were 19.56% ± 0.95%, and the Risser sign which indicates the growth potential was 2.77 ± 1.64. As for the 12 non‐hemivertebra osteotomy patients, the Cobb Angle was 43.62° ± 13.71°, the flexibility ratios were 29.17% ± 13.55%, and the Risser sign was 3.83 ± 1.26.

### Posterior Correction with Fusion

The patient was placed in a prone position with the head fixed by halo traction. A posterior midline incision was made with the main curve in the center to expose the spinous process and lamina layer by layer, and implants of an appropriate size were selected according to the imaging data. Lateral mass screws were inserted into the cervical spine, and pedicle screws were inserted in C6‐7 if the pedicle was wide and without obvious structural malformations.^10^ Pedicle screws were inserted if possible in the thoracic segment; a transverse hook was used if the structure was abnormal and screw insertion was difficult. An upper neutral vertebra was selected as the upper instrumented vertebra (UIV), and the lower instrumented vertebra (LIV) was 1–2 vertebrae below the lower end vertebra (LEV). Ponte osteotomy was performed on the facet joint.^11^ Pre‐shaped correction rods were inserted to finish the correction. If the diameters of the cervical and thoracic correction rods did not match, a connector or a transitional rod was used to achieve a connection. Then, the fused ribs were separated. Intraoperative C‐arm radiography was used to evaluate the position of the internal fixation and determine whether the correction was satisfactory; then, the transverse connection was placed, and repeated flushing with hydrogen peroxide and normal saline was performed. The articular cartilage and cortical bone of the lamina in the fusion segment were removed to make a bone graft bed. Mixed autologous and allograft bone were inserted, and drainage tubes were placed before the incision was closed layer by layer.

### Posterior Hemivertebra Osteotomy

On the basis of the above surgical procedures, the posterior elements of each hemivertebra, including the spinous process, lamina and part of the rib head, were removed at the hemivertebra, the posterior edge of the vertebra was exposed along the medial side of the pedicle by blunt dissection, and the lateral and anterior edges of the hemivertebra were exposed along the lateral side of the pedicle. A stabilizing rod was placed on the concave side before osteotomy. The hemivertebra was removed with a bone knife and other tools; the use of an ultrasonic bone knife can greatly reduce surgical trauma to soft tissue, especially nervous tissue and vascular tissue. For fully segmented hemivertebrae, the upper or lower endplates and intervertebral discs should be removed.^12^ After completion of the osteotomy, the residual cavity was closed until the bone surface was closed, and pre‐shaped correction rods were inserted to complete the correction. The fusion segments were at least two vertebrae above and below the hemivertebra.

### Postoperative Treatment and Follow‐Up

The patients' vital signs, limb activity, sensory function, and other conditions were closely observed after surgery, and X‐ray radiography, CT, and MRI were performed to evaluate the correction rate and exclude postoperative hematoma or other complications. An external fixation support vest was worn beginning 2 weeks after surgery when the patient began to walk and continued to be worn for at least 3–6 months until imaging data indicated complete bone graft fusion. A clinical review was performed once every 3 months postoperatively and once annually after 1 year.

### Evaluation Criteria

**Fig. 1:**
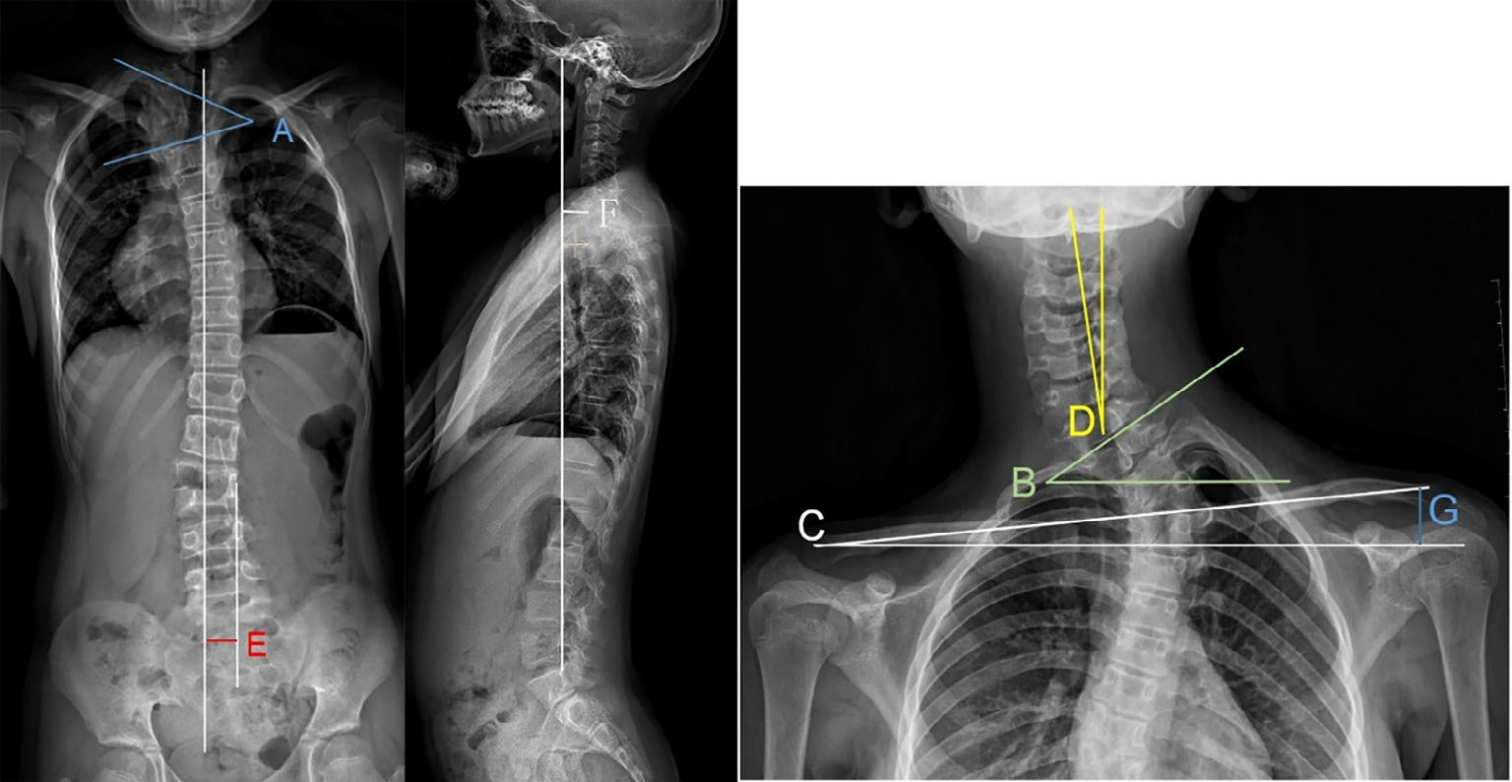
Illustration of parameter measurements on the coronal and sagittal planes. (A) Local Cobb angle: the Cobb angle of the main curve. (B) T1 tilt angle: the angle between the horizontal line and the line through the T1 upper endplate. (C) Clavicular angle: the angle between the horizontal line and the line connecting the two highest points of each clavicle. (D) Neck tilt angle: the angle between the vertical line and the vertical axis of the cervical spine(drawn from the center of the odontoid process of C2 to the center of C7). (E) Coronal balance distance (CBD): the distance between the vertical line through the center of C7 and the center sacral vertical line (CSVL) on the coronal plane. (F) Sagittal vertical axis (SVA): the length of the horizontal line connecting the posterior superior sacral endplate to the vertical line from the center of C7. (G) Radiographic shoulder height (RSH): the vertical distance between the two acromioclavicular joints

Preoperative, postoperative, and last follow‐up measurements were compared. The local Cobb angle was measured to evaluate the size and correction of the main curve; coronal and sagittal trunk balance were measured to evaluate the effect of trunk imbalance correction. The T1 tilt angle, clavicular angle, and radiographic shoulder height (RSH) were used to evaluate shoulder imbalance. The neck tilt angle was used to assess the change in cervical tilt (Figure 1).

### Statistical Analysis

SPSS was used for data analysis. Paired‐sample *t* tests were used to compare the overall preoperative and postoperative data, and independent‐sample *t* tests were used to compare the data of patients in the osteotomy and nonosteotomy groups. Data with normal distribution were reported as mean ± SD, while data with no normal distribution as median (range). *p* < 0.05 was considered statistically significant.

## Results

### Surgical Outcomes of all Patients

A total of 21 patients with CTS were included in this study, nine of whom underwent posterior hemivertebra osteotomy and 12 of whom underwent posterior correction and fusion. The median operation time was 6.5 hours (range, 5–11 hours), and the median blood loss was 700 ml (range, 400–1200 ml).

Twenty‐one patients were followed up for a median of 36 months (range, 18–72 months). The Cobb angle was corrected from 45.81° ± 14.23° preoperatively to 10.48° ± 5.56° postoperatively, with a correction rate of 77.78% ± 8.93%. The T1 tilt angle decreased from 15.26° ± 7.08° preoperatively to 3.33° ± 2.14° postoperatively, with a correction rate of 73.42% ± 21.86%. There was no significant difference between the last follow‐up and postoperative data (*p* < 0.05). The RSH difference was corrected from 1.13 ± 0.74 cm preoperatively to 0.52 ± 0.42 cm postoperatively, with a correction rate of 39.51% ± 35.65%. The clavicular angle improved from 2.52° ± 1.55° preoperatively to 1.16° ± 0.96° postoperatively, with a correction rate of 47.18% ± 35.84% (Table 1).

**TABLE 1:** Correction rate of surgery (mean ± SD)

Indexes	Pre‐operation	Post‐operation	*t* value	*p* value	Correction rate (%)	Last follow‐up
Local scoliosis Cobb angle (°)	45.81 ± 11.98	10.48 ± 5.56	14.25	<0.001	77.77 ± 8.93	9.99 ± 5.16
T1 tilt (°)	15.26 ± 7.08	3.33 ± 2.14	7.54	<0.001	73.42 ± 21.86	3.23 ± 2.02
Clavicular angle (°)	2.52 ± 1.56	1.16 ± 0.96	4.56	<0.001	47.19 ± 35.83	1.33 ± 1.28
Neck tilt (°)	7.14 ± 3.34	2.33 ± 1.16	6.44	<0.001	61.75 ± 22.81	2.71 ± 1.86
Radiographic shoulder height (RSH) (cm)	1.13 ± 0.74	0.52 ± 0.42	4.25	<0.001	39.51 ± 35.69	0.61 ± 0.56
Sagittal vertical axis (SVA) (cm)	2.16 ± 1.72	1.17 ± 0.97	3.10	0.016	50.14 ± 29.62	1.25 ± 1.15
Coronal balance distance (CBD) (cm)	2.35 ± 1.08	0.78 ± 0.63	6.38	<0.001	58.89 ± 43.28	0.82 ± 0.73
Local kyphosis (°)	11.64 ± 7.34	7.71 ± 4.91	2.14	0.045	45.76 ± 22.03	8.46 ± 5.65

### Comparison of Osteotomy Group and Nonosteotomy Group

**Fig. 2:**
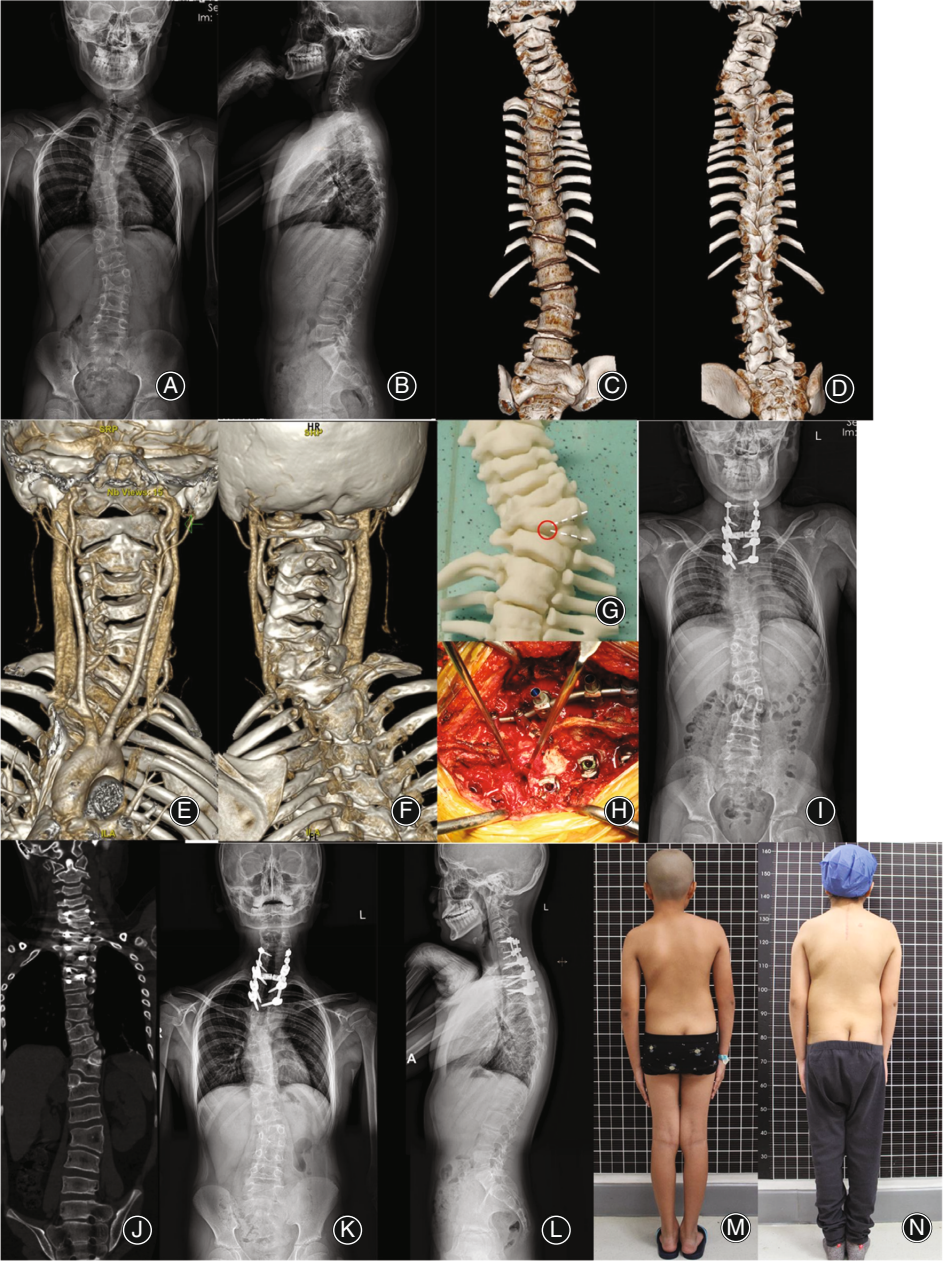
A 12‐year‐old boy with C7 hemivertebra and multiple malformations in the lower spine underwent a C7 hemivertebra osteotomy and internal fixation from C5‐T3 *via* one‐stage posterior surgery. (A, B) Preoperative X‐ray showed a main curve Cobb angle of 54.6° on the coronal plane, with a T1 tilt angle of 12.8°. (C, D, E, F) Preoperative CT and CTA showed a C7 hemivertebra with a large blood vessel around it and multiple malformations in the lower spine. (G, H) The C7 hemivertebra and superior intervertebral disc were resected by posterior approach surgery. (I, J) Postoperative X‐ray showed a main curve Cobb angle of 8.7° with, a correction rate of 87%. CT showed that the bone surface was closed after osteotomy. (K, L) X‐ray at the 2‐year follow‐up showed negligible loss of correction lost. (M, N) Comparison of the preoperative and postoperative appearance showed satisfying correction of the neck tilt and shoulder imbalance

**Fig. 3:**
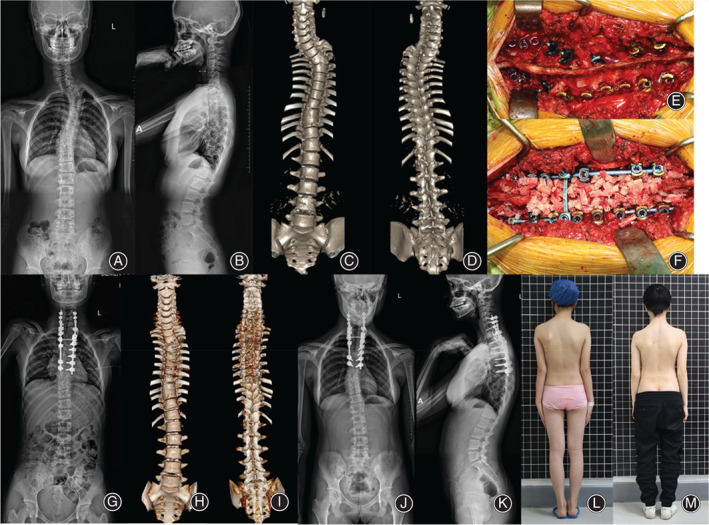
A 13‐year‐old female patient with a T3 hemivertebra underwent posterior correction and fusion surgery without hemivertebra resection. (A, B) Preoperative X‐ray measurement showed a local Cobb angle of 44°,with a T1 tilt angle of 25.3°. (C, D) Preoperative CT showed a T3 hemivertebra with incomplete segmentation. (E, F) A Ponte osteotomy and bone grafting were performed *via* posterior approach surgery. (G) X‐ray immediately postoperatively showed a local Cobb angle of 10.4°, with a correction rate of 76.36%. (H, I) CT showed effective correction without hemivertebra resection. (J, K) Follow‐up X‐ray 3 years after surgery showed satisfactory correction, with no obvious loss of correction. (L, M) The neck tilt improved significantly in appearance after surgery

In addition, 21 patients were divided into an osteotomy group and a nonosteotomy group according to whether a hemivertebra osteotomy was performed, and the data of the two groups were compared. The median blood loss in the osteotomy group was 766 ml (range, 400–1100 ml), and that in the nonosteotomy group was 616 ml (range, 400–1200 ml). The mean operation time in the osteotomy group was 8.4 hours (range, 7–11 hours), while that in the nonosteotomy group was 5.5 hours (range, 4–9 hours). The Cobb angle correction rate was 79.75% ± 9.22% in the osteotomy group and 72.29% ± 8.82% in the nonosteotomy group (Table 2, Figures 2 and 3).

**TABLE 2:** Comparison of the osteotomy group and the nonosteotomy group (mean ± SD)

Indexes	Osteotomy group (*n* = 9)	Nonosteotomy group (*n* = 12)	*t* value	*p* value
Local scoliosis Cobb angle (°)	35.95 ± 6.97	34.85 ± 14.10	0.214	0.883
	79.75 ± 9.22	76.29 ± 8.82	0.872	0.394
T1 tilt (°)	11.01 ± 7.42	12.62 ± 7.36	0.494	0.627
	69.72 ± 25.17	76.20 ± 19.72	0.663	0.551
Clavicular angle (°)	0.49 ± 0.38	1.57 ± 1.48	2.113	0.058
	43.00 ± 33.17	50.32 ± 33.86	0.455	0.654
Neck tilt (°)	5.69 ± 4.60	4.16 ± 2.18	1.014	0.323
	64.09 ± 29.89	60.00 ± 16.97	0.399	0.695
Radiographic shoulder height (cm)	0.51 ± 0.52	0.68 ± 0.75	0.612	0.548
	30.74 ± 19.23	46.10 ± 39.24	0.520	0.649
Sagittal vertical axis (cm)	0.56 ± 0.76	1.73 ± 1.16	2.626	0.017
	24.64 ± 37.26	62.25 ± 26.13	2.378	0.034
Coronal balance distance (cm)	1.33 ± 0.85	1.74 ± 1.31	0.810	0.786
	62.00 ± 28.37	56.56 ± 53.78	0.275	0.439
Local kyphosis (°)	7.26 ± 3.09	8.63 ± 4.93	0.472	0.642
	56.7 ± 22.78	37.98 ± 33.27	0.292	0.774

Note: For each index, the upper line is correction value and the below line is correction rate (%)

### Clinical Function Evaluation

All patients had completed the SRS‐22 questionnaires before surgery and at the last follow‐up. The SRS‐22 questionnaires contain five domains: function, pain, self‐perceived image, satisfaction with treatment, and mental health. Among them, self‐perceived image and satisfaction with treatment showed improvement at the last follow‐up, while the other three domains maintained almost the same since no significant symptoms were observed before surgery and at the last follow‐up (Table 3).

**TABLE 3:** Score of SRS‐22

Indexes	Pre‐operation	Last follow‐up	*t* value	*p* value
Pain	4.6 ± 0.2	4.8 ± 0.1	1.23	0.21
Function	4.7 ± 0.2	4.8 ± 0.2	0.49	0.33
Self‐image	3.1 ± 0.6	4.6 ± 0.3	3.09	<0.01
Mental health	4.6 ± 0.4	4.7 ± 0.2	0.31	0.18
Satisfaction	3.7 ± 0.5	4.8 ± 0.2	2.82	<0.01
Total	4.2 ± 0.6	4.8 ± 0.3	2.53	<0.02

### Complications

One of the patients experienced transient upper limb numbness after C7 hemivertebra osteotomy, which returned to normal after conservative treatment including low dose glucocorticoids and neuronutrition therapy for 2 weeks, while the other 20 patients experienced no obvious postoperative complications. No fixation failure was observed at the last follow‐up.

## Discussion

CTS is uncommon and quite unique in spinal deformities. All the 21 patients in the study achieved good treatment results without serious postoperative complications. By analyzing the preoperative and postoperative data, we compared correction effects of the two surgical methods and try to explore the indication of different surgeries.

### Characteristics of CTS

CTS refers to deformities of the spine with the apex of the scoliosis located at C6‐T4.^1^, ^2^ The main cause of the disease is congenital deformity. The incidence of congenital CTS is low, accounting for approximately 1%–2% of congenital scoliosis cases. Such patients can occur multiple hemivertebrae or incomplete segmentation of other segments.^13^ In this study, 12 patients with other segmental malformations did not exhibit an obvious appearance of the malformations due to the small angle and good flexibility, and no special treatment other than observation was given.

The junction of the cervical and thoracic spine has a complex^14^ anatomical structure and is adjacent to important nerve vessels and organs, and surgery in this area frequently causes injury and leads to serious complications. Therefore, routine preoperative CT, CTA, and MRI examinations are performed to determine the positional relationship of neurovascular structures and spinal cord malformations,^15^, ^16^ such as split cord malformation (SCM) and syringomyelia. Intraoperative electromyography (EMG) can show changes in nervous system function in real time,^17^ indicating stimulation of the cord by the operation, and can be used to effectively avoid injury to the spinal cord. The vertebral artery generally enters the transverse foramen of C6, and congenital spinal malformations are often associated with vascular abnormalities. Therefore, preoperative arterial CTA was routinely performed to observe any vertebral artery deformities. Failure of internal fixation is a frequently reported complication. The difficulty of screw placement in the cervicothoracic spine is greatly increased by factors such as narrow pedicles and abnormal vertebral structures.^18^ Preoperative spinal CT can show the structure of spinal deformities and the size of the pedicle, and individualized surgical plans can be made according to different patient conditions, such as the position and depth of screw placement. 3D printing can be used to display the shape of the spine more intuitively, which is helpful for surgical planning and as an intuitive reference during surgery. Intraoperative C‐arm radiography is routinely used to determine whether the screws are in a good position. Due to a high level of cervical activity, we emphasize the long‐term postoperative use of external fixation support for at least 3 months to decrease the risk of internal fixation failure. Removing the intervertebral disc of fully segmental hemivertebrae can eliminate growth potential, preventing loss of correction after surgery.

### Analysis of Surgical Outcomes

In this study, comparison between the osteotomy and nonosteotomy groups showed that the surgical duration (5.5 ± 0.67 h) and blood loss (616.66 ± 224.95 ml) of posterior fusion surgery were significantly lower without than with osteotomy (8.44 ± 1.23 h, 766.66 ± 244.94 ml). This is related to the complexity of the operation and extent of surgical trauma. Comparison of the corrective effect showed similar correction rates of the local Cobb angle (79.75% ± 9.22% in the osteotomy group and 76.29% ± 8.82% in the nonosteotomy group), and the two groups had similar results in terms of correction of the clavicular angle, T1 tilt angle, RSH difference, and cervical angle (*p* > 0.05). For patients requiring osteotomy with poor flexibility (19.56% ± 9.95%), greater correction (30°–40°) was achieved by osteotomy of a single hemivertebra, while patients with good flexibility (29.42% ± 13.48%) benefited from direct correction surgery and had longer fusion segments, with results similar to those in the osteotomy patients. Since the cases collected in this paper were cases of cervical thoracic lateral curvature deformities, the distance between the cervical thoracic kyphosis angle and the point of sagittal balance was mostly within the normal range, and no significant changes were observed after orthopaedic surgery. Therefore, the data obtained from the measurement of sagittal balance and the local kyphosis angle before and after surgery and at the last follow‐up were not significantly different.

### Posterior Correction with Fusion

For patients with good flexibility and compensatory cervical vertebrae, we applied posterior correction with fusion, which can achieve the same orthopaedic effect while avoiding hemivertebra osteotomy. Since the hemivertebra was not removed, the fixed segment was lengthened accordingly to achieve a satisfactory orthopaedic effect, with the UIV fused to the upper neutral vertebra and the LIV fused to one or two vertebrae below the LEV. For patients with fused ribs,^19^ releasing the fused ribs can ease extension on the concave side. C‐arm X‐ray should be repeated during the operation to clarify the orthopaedic status, and excessive orthopaedic coverage should not be avoided.

### Hemivertebra Osteotomy

For patients with poor flexibility and severe deformities, one‐stage posterior correction cannot achieve satisfactory results. Posterior osteotomy and fusion can be applied to increase the correction rate. It is recommended that the fusion level be fixed to at least two segments above and below the hemivertebra. During the osteotomy, attention should be given to reducing the destruction of bone in the screw placement area to decrease the difficulty of screw placement. For those requiring a hemivertebra osteotomy at the segment above T1, combined anterior and posterior approach surgery has been recommended in the literature.^5^, ^6^ Combined anterior and posterior approach surgery can be used to perform a hemivertebra osteotomy *via* the anterior approach. The advantage of this approach is that the surgeon is familiar with the anterior approach, and the operative field is clear, which improves the safety of anterior hemivertebra osteotomy. However, the combined anterior and posterior approach not only increases the surgical trauma and the duration of the operation but also challenges patients and surgeons by requiring intraoperative changes in position. Thus, we recommend the use of individualized surgical plans that consider the specific structure of the cervicothoracic segment shown by preoperative imaging data. Two patients with hemivertebrae above T1 were included in this study, one with a C7 hemivertebra and the other with a C6 hemivertebra. No anterior surgery was performed. The C6 hemivertebra patient did not undergo a hemivertebra osteotomy but underwent posterior correction with fusion only. For the patient with the C7 hemivertebra, the preoperative imaging data showed that the vertebral artery did not enter the transverse foramen at the hemivertebra; thus, we decided to resect the hemivertebra *via* posterior approach surgery, which resulted in a satisfying corrective effect. We believe that for patients with a C7 hemivertebra requiring osteotomy and orthopaedic surgery, if CTA and MRI show no important nerves or blood vessels near the osteotomy area, correction can be achieved by one‐stage posterior osteotomy, which greatly reduces the operation time, blood loss, and trauma and does not require repeated intraoperative changes in position, thus effectively reducing intraoperative and postoperative complications.

### Limitations and Strengths

There are some limitations that should be considered. First, the sample size of included patients is not big enough. Second, the medium‐ and long‐term follow‐up results require further evaluation. The advantage is that we performed a detailed analysis of a number of CTS patients who have achieved satisfactory results through both surgeries, and provide some guidance for the selection of surgical methods.

### Conclusion

One‐stage posterior correction surgery with fusion and one‐stage posterior hemivertebra osteotomy can be used to treat CTS and obtain satisfactory results. Whether hemivertebra osteotomy was necessary should be determined according to different conditions including the main curve angle, flexibility, and growth potential. Further research can focus on enlarging the sample size, which may provide a more precise and detailed criterion of surgical method selection through data analysis.

## Disclosure

The authors declare that they have no competing interests and do not have any financial support or relationship that may pose conflict of interests.

## Supporting information


**Table S1.** Basic information
