# Supplementary material for: Strategy and Efficacy of Surgery for Congenital Cervicothoracic Scoliosis with or without Hemivertebra Osteotomy
Source: Orthop Surg. 2022 Aug 30;14(9):2050–8. doi: 10.1111/os.13480 (PMC9483056; doi:10.1111/os.13480)
Supplement: Supplementary file 1 — Table S1. Basic information [file OS-14-2050-s001.docx]

**Supplemental Table.** Basic information

| **ID** | **Age(yrs)** | **Sex** | **Cobb Angle(°)** | **Flexiblity（%）** | **Risser**  **Sign** | **Type of deformity** | **Hemivertebra**  **osteotomy** |
| --- | --- | --- | --- | --- | --- | --- | --- |
| **1** | 14 | male | 22.40 | 33 | 5 | T3 hemivertebra | no |
| **2** | 13 | female | 73.20 | 23 | 4 | C2-4 block vertebrae, T4 hemivertebra | no |
| **3** | 11 | female | 66.90 | 11 | 2 | T3 hemivertebra, T4-5 fused ribs | yes |
| **4** | 14 | male | 50.20 | 15 | 3 | T4 hemivertebra, T4-5 block vertebrae | yes |
| **5** | 19 | female | 45.80 | 27 | 5 | T4 hemivertebra | yes |
| **6** | 12 | male | 54.60 | 19 | 2 | Multiple block vertebrae and hemivertebrae, C7 hemivertebra | yes |
| **7** | 7 | female | 37.60 | 4 | 0 | T2, T8 hemivertebrae | yes |
| **8** | 13 | female | 34.00 | 10 | 4 | T1 butterfly vertebra, T3 hemivertebra | no |
| **9** | 15 | female | 64.10 | 41 | 4 | Multiple block vertebrae and hemivertebrae | no |
| **10** | 9 | female | 36.20 | 19 | 1 | T3 hemivertebra with incomplete segmentation | yes |
| **11** | 13 | male | 43.10 | 63 | 5 | Multiple hemivertebrae | no |
| **12** | 11 | female | 41.10 | 28 | 3 | T4 hemivertebra | no |
| **13** | 14 | male | 48.20 | 30 | 4 | C7, T8 hemivertebrae | yes |
| **14** | 14 | female | 49.50 | 31 | 5 | T1-3 block vertebrae | no |
| **15** | 16 | male | 47.00 | 36 | 4 | T3 hemivertebra | yes |
| **16** | 14 | female | 39.80 | 19 | 4 | T4 hemivertebra and incomplete segmentation | no |
| **17** | 15 | female | 44.60 | 30 | 5 | T3 hemivertebra, T9 hemivertebra with incomplete segmentation | no |
| **18** | 13 | female | 34.70 | 16 | 4 | T4 hemivertebra | no |
| **19** | 14 | male | 52.10 | 15 | 4 | T2 hemivertebra | yes |
| **20** | 9 | female | 43.00 | 27 | 2 | T2 hemivertebra T5 butterfly vertebrae, incomplete segmentation | no |
| **21** | 8 | female | 34.00 | 32 | 1 | T4 hemivertebra | no |

**This study has been approved by the Ethics Committee of Xiangya Hospital, Central South University (ethics approval number: 201703359).**
